# Title: Hypermethylation of miRNA Genes During Nodule Development

**DOI:** 10.3389/fmolb.2021.616623

**Published:** 2021-04-13

**Authors:** Sarbottam Piya, Valeria S. Lopes-Caitar, Won‐Seok Kim, Vince Pantalone, Hari B. Krishnan, Tarek Hewezi

**Affiliations:** ^1^ Department of Plant Sciences, University of Tennessee, Knoxville, TN, United States; ^2^ Plant Science Division, University of Missouri, Columbia, MO, United States; ^3^ Plant Genetics Research, USDA-Agricultural Research Service, Columbia, MO, United States

**Keywords:** DNA methylation, epigenetics, gene expression, miRNA, nitrogen-fixing nodules, soybean (*Glycine max*)

## Abstract

DNA methylation has recently emerged as a powerful regulatory mechanism controlling the expression of key regulators of various developmental processes, including nodulation. However, the functional role of DNA methylation in regulating the expression of microRNA (miRNA) genes during the formation and development of nitrogen-fixing nodules remains largely unknown. In this study, we profiled DNA methylation patterns of miRNA genes during nodule formation, development, and early senescence stages in soybean (*Glycine max*) through the analysis of methylC—seq data. Absolute DNA methylation levels in the CG, CHH, and CHH sequence contexts over the promoter and primary transcript regions of miRNA genes were significantly higher in the nodules compared with the corresponding root tissues at these three distinct nodule developmental stages. We identified a total of 82 differentially methylated miRNAs in the nodules compared with roots. Differential DNA methylation of these 82 miRNAs was detected only in the promoter (69), primary transcript region (3), and both in the promoter and primary transcript regions (10). The large majority of these differentially methylated miRNAs were hypermethylated in nodules compared with the corresponding root tissues and were found mainly in the CHH context and showed stage-specific methylation patterns. Differentially methylated regions in the promoters of 25 miRNAs overlapped with transposable elements, a finding that may explain the vulnerability of miRNAs to DNA methylation changes during nodule development. Gene expression analysis of a set of promoter-differentially methylated miRNAs pointed to a negative association between DNA methylation and miRNA expression. Gene Ontology and pathways analyses indicate that changes in DNA methylation of miRNA genes are reprogrammed and contribute to nodule development through indirect regulation of genes involved in cellular processes and pathways with well-established roles in nodulation.

## Introduction

The formation of nitrogen fixing nodules in plant roots is a host-specific process that involves well-established chemical communication signals between host plants and Rhizobia (Verma, 1992; Wang et al., 2018). Once receiving the chemical signal from host plants, the compatible nitrogen-fixing bacteria activate the expression of nodulation genes and produce nodulation factors (Nod factors) (Singla and Garg, 2017; Altúzar-Molina et al., 2020). Recognition of the Nod signal by the host plants triggers an array of morphological, physiological, and structural changes in root cells leading to the formation of nodules (Stubbendieck et al., 2019). These changes are accompanied and mediated by massive changes in the expression of thousands of genes (Quist et al., 2015; Mergaert et al., 2020). Transcription factor of the GRAS, AP2/ERF, NAC, NF-Y, bZIP, C2H2, bHLH, MYB, and WRKY families are believed to be the key regulators responsible for gene expression changes during nodule differentiation, formation, and development (Schauser et al., 1999; Soyano and Hayashi, 2014; Kawaharada et al., 2017; Liu et al., 2020; Niyikiza et al., 2020; Tan et al., 2020). Also, microRNA (miRNA) genes have been shown to play key regulatory functions during nodulation (Hoang et al., 2020).

MicroRNAs (miRNAs) are short (21–22 nt) non-coding RNA molecules, which negatively regulate the expression of their target genes containing a complementary binding sites through mRNA degradation or translational repression (Treiber et al., 2019). Functional characterization of a significant number of miRNA genes in various plant species revealed their broad regulatory functions ranging from cellular differentiation and organ development to responses to biotic and abiotic stresses. The advent of small RNA sequencing technology together with the development of bioinformatic tools resulted in the identification of miRNA genes from various plant tissues and developmental organ including root nodules (Zilberman et al., 2007; Turner et al., 2012; Song et al., 2015; Yan et al., 2015, 2016). Furthermore, degradome sequencing, a modified 5′-rapid amplification of cDNA ends (RACE) method, allowed the determination of cleavage sites of miRNAs, and hence their target genes at a large scale (Song et al., 2011; Fang et al., 2013; Arikit et al., 2014; Yan et al., 2015, 2016; Zhao et al., 2015; Ding et al., 2016).

Analysis of miRNA expression patterns in the nodules revealed that many miRNAs are expressed in a tissue and stage—specific fashion (Tsikou et al., 2018; Reynoso et al., 2019; Tiwari and Bhatia, 2020). Nevertheless, the mechanisms controlling the spatiotemporal expression patterns of miRNAs during nodulation are largely unknown. In this context, DNA methylation as a highly dynamic and reversal epigenetic mark has the potential together with genetic mechanisms to regulate miRNA expression in the developing nodules. In plants, DNA methylation exist in symmetric (CG and CHG) and asymmetric (CHH) sequence contexts, which are established with specific methyltransferases (Wendte and Schmitz, 2018; Zhang et al., 2018). The symmetric CG and CHG methylation contexts are maintained by METHYLTRANSFERASE1 (MET1), and CHROMOMETHYLASE3, respectively, (Finnegan and Kovac, 2000; Lindroth, 2001; Kankel et al., 2003). The asymmetric CHH methylation context is maintained by CMT2, DOMAINS REARRANGED METHYLTRANSFERASE2 (DRM2), and DRM3 via the RNA-directed DNA methylation (RdDM) pathway, which is also required for *de novo* DNA methylation in all sequence contexts (Cao and Jacobsen, 2002; Zhang and Zhu, 2011; Matzke and Mosher, 2014; Stroud et al., 2014).

The role of DNA methylation as transcriptional silencing mark of protein-coding genes and transposable elements (TEs) is well-established both in plants and animals (Zhang et al., 2018; de Mendoza et al., 2020). Genome—wide DNA methylation analysis during various stages of nodule development in *Medicago truncatula* revealed widespread DNA hypomethylation patterns particularly in the CG and CHG sequence contexts (Satgé et al., 2016). Additional experimental evidence revealed the key role of MtDME in establishing the hypomethylation patterns in immature and fully developed nodules that impact the expression of a significant number of genes with functions related to symbiotic nodule development (Satgé et al., 2016). Also, symbiotic islands identified in the recently sequenced *M. truncatula* A17 genome displayed differential patterns of DNA methylation and histone marks when nodule and root tissues were compared (Pecrix et al., 2018). In contrast to *M. truncatula*, genome-wide analysis of DNA methylation profiles in three nodule developmental stages in soybean revealed global increases in DNA methylation levels in all sequence contexts as compared with corresponding root tissues (Niyikiza et al., 2020). Identification of the differentially methylated regions and the overlapping genes pointed to a major role of DNA methylation in regulating gene expression and alternative splicing events during nodule differentiation and development (Niyikiza et al., 2020).

The role of DNA methylation in controlling transcription and biogenesis of non-coding genes such as miRNAs was also demonstrated in mammalian cell systems (Glaich et al., 2019). However, limited experimental evidence in plants suggests that DNA methylation of miRNA genes is of biological significance. For example, 113 methylated miRNA genes were identified during methylome analysis of male and female flowers in andromonoecious poplar (*Populus tomentosa*) (Song et al., 2015). Gene expression analysis and target identification of these methylated miRNAs pointed to a key role of DNA methylation in bisexual flower development (Song et al., 2015). Biotic and abiotc stresses can also induce functional DNA methylation changes in miRNA genes. For example, in *Populus simonii*, heat, osmotic, cold, and salinity stress treatments were found to induce hyper- and hypomethylation of miRNAs that impacted miRNA gene expression, and subsequently the expression of target genes (Song et al., 2016). In soybean, infection by soybean cyst nematode has been shown to trigger substantial alteration in DNA methylation patterns of miRNA genes during both compatible and incompatible interactions (Rambani et al., 2020a). Functional characterization of a set of the differentially methylated miRNAs revealed their role in determining soybean resistance to nematode infection (Rambani et al., 2020a).

In this study, we analyzed global DNA methylation patterns of miRNA genes during three discrete stages of nodule development in soybean and identified miRNA genes whose DNA methylation levels were significantly altered in nodules compared with roots. We found the majority of the differentially methylated miRNAs were hypermethylated predominantly in the CHH sequence context. We detected positive correlation between miRNA methylation and the expression of target genes; many of them are involved in biological processes and pathways associated with nodule formation and development. Together, our results point to an important role of DNA methylation as a regulatory mechanism of miRNA genes during soybean nodulation.

## Materials and Methods

### Bacterial Strain and Growth Conditions

*Bradyrhizobium diazoefficiens* (strain USDA110) cells were cultured in 30 ml of yeast extract-mannitol (YEM) broth containing spectinomycin at a final concentration of 100 μg/ml with shaking at 150 rpm at 30°C for 3 days. This preculture was used to inoculate 1 L of YEM broth medium without antibiotic. The cultures were grown at the same conditions for additional three days and then used to inoculate germinating soybean seeds as described below.

### Plant Growth Conditions and Tissue Collection

Soybean [*Glycine max* (L.) Merr. cv Williams 82] seeds were surface-sterilized with 2.5% sodium hypochlorite (NaClO). Roughly, two hundred surface-sterilized soybean seeds were germinated in plastic trays (50 × 28 × 15 cm) containing vermiculite. Each tray was watered with 4 L sterile water at the time of planting. Three days after germination in the dark, the *B. diazoefficiens* cultures were diluted in YEM broth to a final concentration of 4.5 × 10^6^ cells/ml and were poured on the top of each tray. The inoculated trays were transferred to a growth chamber and were incubated at 28°C under a photoperiod of 12 h of light and 12 h of dark and light intensity of 500 µmol of photons m^2^ sec^−1^. Non-inoculated trays were grown under the same conditions and used as control treatments. At 12, 22, and 36 days after inoculation (dpi), nodules and the corresponding root tissues were collected in three biological replicates and used for RNA extraction.

### Identification of Differentially Methylated Regions Overlapping With miRNA Genes

A total of 18 methylC-seq libraries, constructed from three biological samples of DNA isolated from nodules at 12, 22, and 36 dpi and the corresponding control root tissues (Niyikiza et al., 2020) were analyzed in this study. Low quality reads and adapters of methylC-seq data were removed with trimmomatic, version 0.35 (Bolger et al., 2014). High quality reads of each library were mapped to the soybean genome (*Glycine*_max_v2.0; http://https://www.ncbi.nlm.nih.gov/) separately using Bismark (Krueger and Andrews, 2011). The annotation of soybean miRNA genes was retrieved from miRBase database (http://www.mirbase.org/). Cytosine to uracil conversion rate was determined by spiking the unmethylated lambda phage DNA to all nodule and root samples before treatment with sodium bisulfite. R package Methylkit (Akalin et al., 2012) was used to identify methylated cystosines for each library in the CG, CHG, and CHH sequence contexts in nodules and control roots at 12, 22, and 36 dpi. Cytosines were considered for identifying differentially methylated regions (DMRs) only if they were covered by at least 10 high-quality reads. DMRs between nodules and the corresponding roots were identified using of 200 bp non-overlapping windows. Regions with at least 25% difference in cystosine methylation with an adjusted *p*-value of less than 0.01 were considered as differentially methylated. DMRs were overlapped with the promoter and the primary transcript regions of the miRNAs. miRNA genes overlapping with at least one DMRs were considered as differentially methylated. The 2 kb region upstream of miRNA transcriptional start site was considered as promoter. Bedtools (Quinlan and Hall, 2010) was used to identify overlaps between differentially methylated miRNAs and TEs. Genome-wide DNA methylation profiles were generated using ViewBS package with absolute cystosine methylation data (Huang et al., 2018). Boxplots of absolute cystosine methylation levels were generated using the R package ggplot2 Heatmaps representing methylation levels of DMRs overlapping with miRNAs were generated using the R package pheatmap.

### MicroRNA Target Prediction

The psRNATarget server (http://plantgrn.noble.org/psRNATarget/) (Dai et al., 2018) was used to predict putative targets of the differentially methylated miRNAs. A highly stringent penalty score of 3.0 or lower (Rambani et al., 2020a) was applied as threshold for predicting the miRNA targets.

### Gene Ontology and KEGG Pathway Enrichment Analysis

All target genes of differentially methylated miRNAs were mapped to GO terms in the GO databases (http://www.geneontology.org/). Statistically significantly enriched GO terms in the list of target genes of differentially methylated miRNAs compared with genome background were determined using Fisher’s exact test with a significance cutoff *p*-value of 0.05. KEGG analysis was performed using KOBAS (Xie et al., 2011) with Fisher’s exact test and Benjamini and Hochberg false discovery rate (FDR) < 0.05 for significance.

### Quantitative Real-Time RT-PCR of miRNAs and Target Genes

Total RNA was extracted using TRIzol reagent (Invitrogen) according to the manufacturer’s instructions. cDNA was synthesized through reverse transcription of total RNA using Mir-X miRNA First-Strand Synthesis Kit (Clontech) according to the manufacturer’s instructions. qRT-PCR was performed using PerfeCTa™ SYBR® Green FastMix™ following manufacturer’s instructions. Reaction mix comprised of 5 µL of Green FastMix [2x], 1 µL each of forward miRNA specific primer (10 µM) and mRQ 3′ primer [10 µM], 50 ng of cDNA and MilliQ water to a 10 µL final volume. The PCR program was 95°C for 3 min followed by 40 cycles of 95°C for 30°s and 65°C for 30°s. Amplification specificity was determined by generating dissociation curves. The dissociation program was 95°C for 15°s and 50°C for 15°s, followed by a slow gradient from 50 to 95°C. qRT-PCR reactions were carried out in QuantStudio six Flex Real-Time PCR System (Applied Biosystems). Relative expression was calculated as previously described by Livak and Schmittgen (2001). Small nuclear RNA U6 and 60°S (*Glyma.13G318800*) genes were used as internal control to normalize gene expression data. Primer sequences used in qRT-PCR assays are provided in Supplementary Table S8.

### Accession Numbers

MethylC-seq and RNA-seq data used in this study can be found in Gene Expression Omnibus (GEO) database repository under accession number GSE135972.

## Results

### Global Hypermethylation of miRNA Genes Occurs During Nodule Development

**FIGURE 1:**
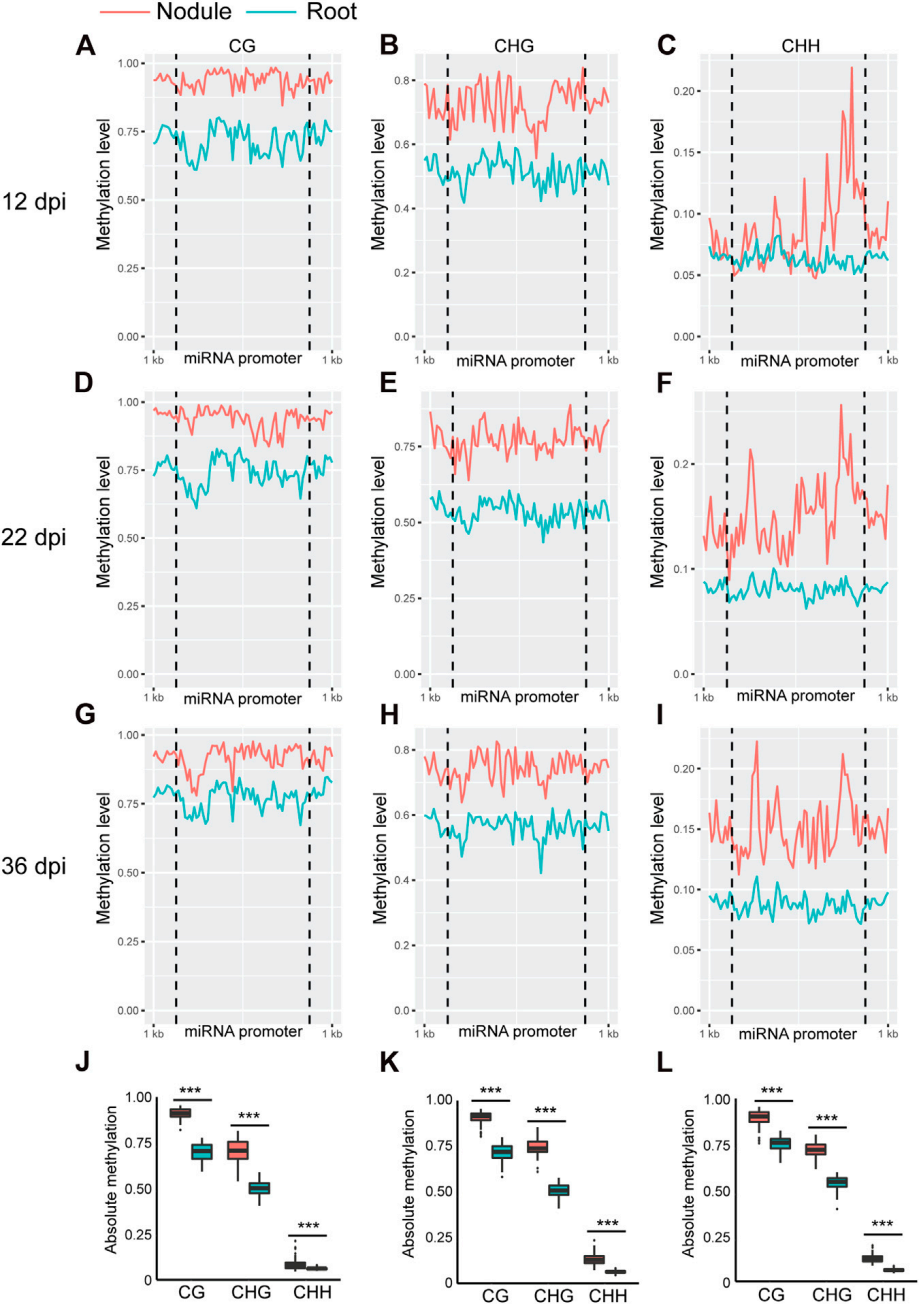
Absolute DNA methylation levels across miRNA genes in soybean nodules and roots at 12, 22, and 36 dpi. **(A–I)**: Differences in global DNA methylation levels between nodules and roots over miRNA genes in the CG, CHG, and CHH sequence contexts at 12 **(A–C)**, 22 **(D–F)**, and 36–dpi **(G–I)**. The vertical dotted lines flank the prompter region, and 1 kb indicates the distance from the middle of miRNA promoter. **(J–L)**: Boxplots demonstrating statistically significant differences in absolute methylation levels across miRNA genes between nodules and roots in various sequence contexts at 12 **(J)**, 22 **(K)** and 36 dpi **(L)**. Asterisks indicate *p* < 0.001 for the comparison between nodules and roots as determined by *t* test.

**FIGURE 2:**
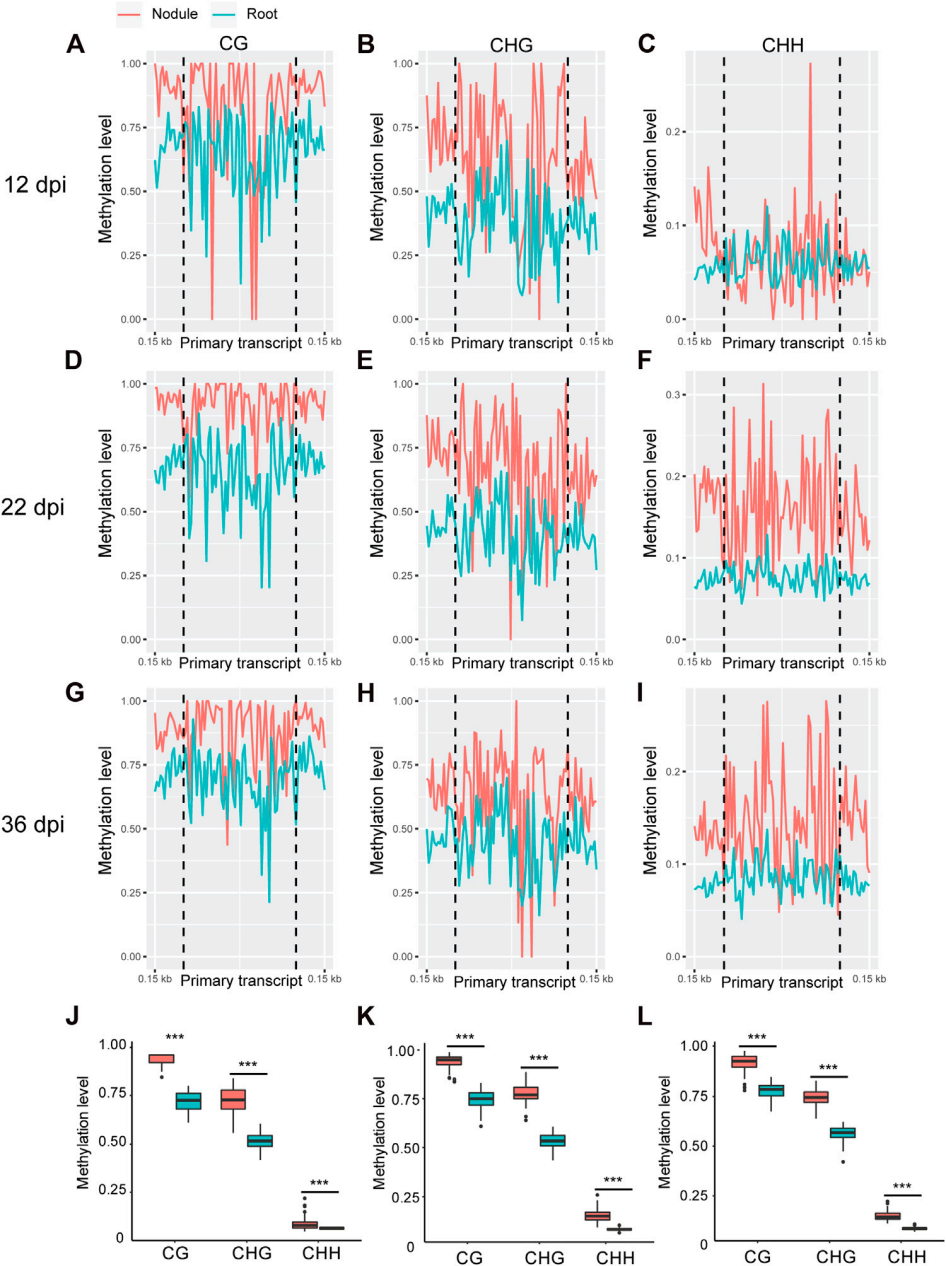
Absolute DNA methylation levels across miRNA primary transcript region in soybean nodules and roots at 12, 22, and 36 dpi. **(A–I)**: Differences in global DNA methylation levels between nodules and roots over miRNA primary transcript region in the CG, CHG, and CHH sequence contexts at 12– **(A–C)**, 22– **(D–F)**, and 36–dpi **(G–I)**. The vertical dotted flank the miRNA primary transcript region, and 0.15 kb indicates the distance from the middle of primary transcript region. **(J–L)**: Boxplots demonstrating statistically significant differences in absolute methylation levels across miRNA primary transcript region between nodules and roots in various sequence contexts at 12 **(J)**, 22 **(K)**, and 36 dpi **(L)**. Asterisks indicate *p* < 0.001 for the comparison between nodules and roots as determined by *t* test.

Whole genome bi-sulfite sequencing reads of nodules collected at 12, 22, and 36 days post inoculation (dpi) with *Bradyrhizobium diazoefficiens* (strain USDA110) and the corresponding root tissues (Niyikiza et al., 2020) were mapped to the promoter and primary transcript regions of all the known miRNA genes in the soybean genome (NCBI_Assembly: GCA_000004515.3). These three time points correspond to nodule formation, development, and early senescence stages. The nodule formation stage is characterized by active cell division where nitrogen fixation starts. During nodule development stage, the nodules reach their maximize size and nitrogen fixation is at its peak, whereas at the early senescence stages there is a significant decrease in nitrogen fixation (Niyikiza et al., 2020). Cytosine to uracil conversion rate was determined using unmethylated lambda phage DNA and found to be more than 99.9%. Absolute levels of cytosine methylation in the CG, CHG, and CHH contexts were determined over the promoter and primary transcript regions of all annotated soybean miRNA genes. Increases in absolute DNA methylation levels over the promoter region of miRNA genes were detected in the nodules in comparison with the corresponding root tissues. These increases were observed in all sequence contexts at 12 dpi (Figures 1A–C), 22 dpi (Figures 1D–F), and 36 dpi (Figures G–I). Boxplot analysis revealed that the levels of absolute DNA methylation are statistically significantly higher in the nodules compared with root tissues in all sequence contexts at the three time points (Figures 1J–L). Analyzing the absolute levels of cytosine methylation over the primary transcript regions of miRNA genes also revealed higher levels of DNA methylation in the nodules compared with roots in all sequence contexts at the three time points (Figures 2A–I). These increases were statistically significant as determined by boxplot analysis (Figures 2J–L). These results point to a global augmentation in DNA methylation over miRNA genes during nodule formation, development, and early senescence stages.

### Identification of Differentially Methylated miRNA Genes in Developing Soybean Nodules

**FIGURE 3:**
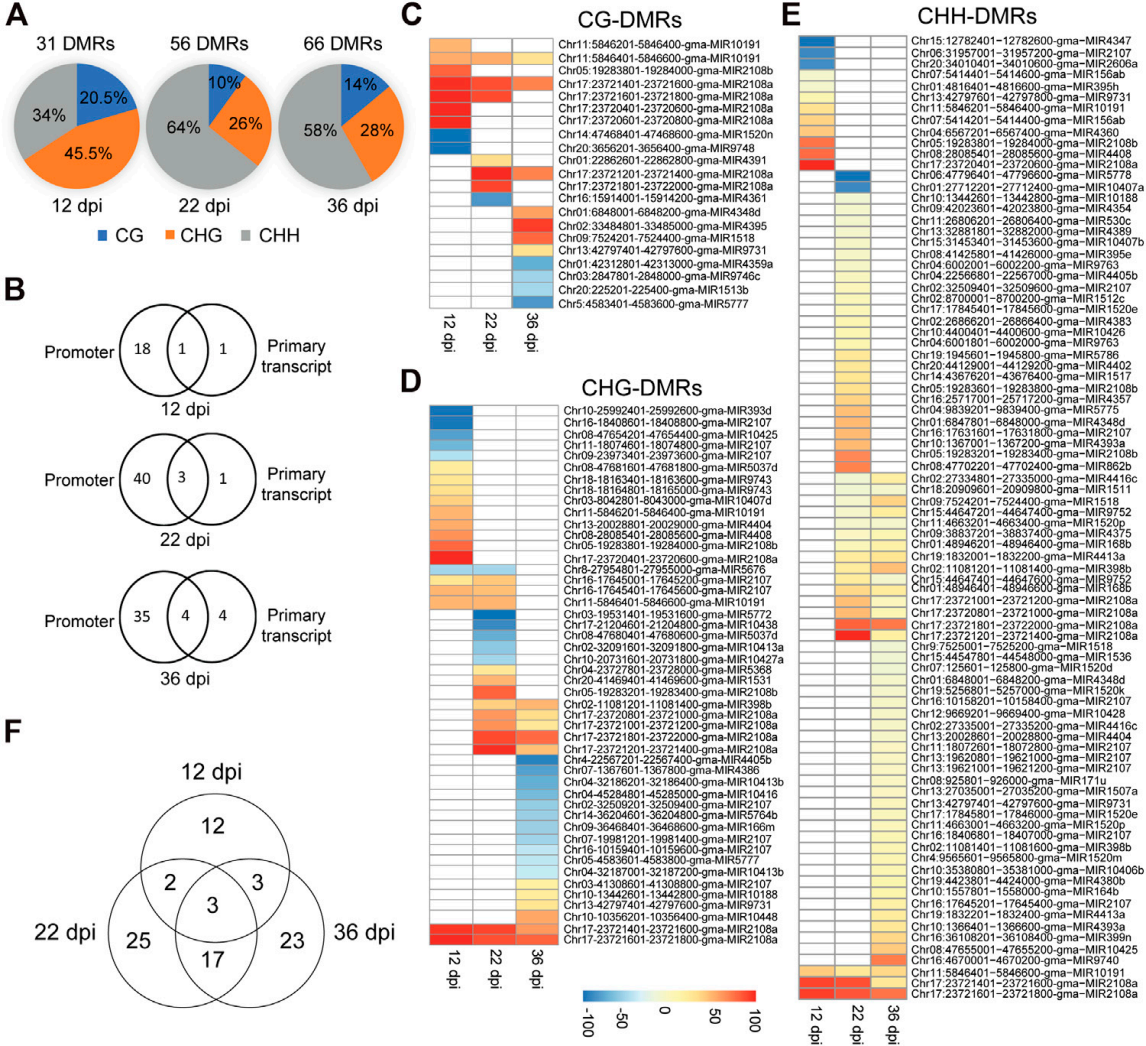
Classification of differentially methylated miRNAs in soybean nodules. **(A)**: Distribution of DMRs identified at 12, 22, and 36 dpi over the CG, CHG and CHH sequence contexts. **(B)**: Number of differentially methylated miRNAs in the promoter or primary transcript regions at 12, 22, and 36 dpi. **(C–E)**: Heatmap demonstrations of hyper-and hypomethylated miRNAs in the nodules as compared with root tissues at 12, 22, and 36 dpi in the CG **(C)**, CHG **(D)**, and CHH **(E)** sequence contexts. Note that some miRNAs contain more than one DMR, which could be differentially methylated in different sequence contexts. **(F)**: Venn diagram showing the overlaps between differentially methylated miRNAs identified at 12, 22, and 36 dpi.

In order to identify soybean miRNAs that undergo significant changes in DNA methylation in nodules compared to the root tissues, we identified differentially methylated cytosines and regions between nodule and root tissues in the promoter and primary transcript regions of miRNAs using 200-basepair non-overlapping windows. A region was considered differentially methylated if showed a minimum of 25% methylation difference between nodules and roots in the CG, CHG, or CHH contexts with a false discovery rate (FDR) less than 1%. A total of 31, 56, and 66 differentially methylated regions (DMRs) were identified at 12, 22, and 36 dpi, respectively, (Figure 3A). The 12-dpi DMRs were found in CG (20.5%), CHG (45.5%), and CHH (34%) (Figure 3A). The 22-dpi DMRs were found in CG (10%), CHG (25.7%), and CHH (64.3%) (Figure 3A). The 36-dpi DMRs were found in CG (13.9%), CHG (27.9%), and CHH (58.2%) (Figure 3A). This analysis shows a substantial increase in the number of CHH-DMRs at 22 and 36 dpi. The DMRs were mapped to the promoter and primary transcript regions of the corresponding miRNAs. A total of 20, 44, and 43 unique miRNAs overlapped with the DMRs at 12, 22, and 36 dpi, respectively, (Figures 3B–E; Supplementary Tables S1–S8) and therefore considered as differentially methylated miRNA genes between nodule and root tissues. The occurrence of DMRs was more prevalent in the promoter than primary transcript region (Figure 3B). We identified 18, 40, and 35 differentially methylated miRNAs only in the promoter region at 12, 22, and 36 dpi, respectively, (Figure 3B), whereas only 1, 1, and 4 miRNAs were identified as differentially methylated only in the primary transcript region at these three time points, respectively (Figure 3B). Also, we found that 1, 3, and 4 miRNAs were differentially methylated between nodule and root tissues both in the promoter and primary transcript regions at 12, 22, and 36 dpi, respectively (Figure 3B).

### Nodule Differentially Methylated miRNAs are Predominantly Hypermethylated in the CHH Context

Differentially methylated miRNAs between nodule and root tissues were further classified into hyper- and hypomethylated for each sequence contexts. Consistent with the global DNA methylation profiles, we found that the numbers of nodule hypermethylated miRNAs (60) are higher than hypomethylated miRNAs (26) when compared with the corresponding root tissues. Four miRNAs showed both hypermethylated and hypomethylated but in different regions. The 60 hypermethylated miRNAs overlapped with 121 regions, which were found in CG (14; 11.6%), CHG (26; 21.5%) and CHH (81; 66.9%) contexts (Figures 3C–E). The 26 hypomethylated miRNAs overlapped with 36 regions, which were found in the CG (8; 22.2%), CHG (23; 63.9%), and CHH (5; 13.9%) contexts (Figures 3C–E). Of note is that some of the DMRs overlapping with hyper and hypomethylated miRNAs were found in more than one sequence context (Supplementary Tables S1–S3). These data indicate that nodule differentially methylated miRNAs are predominantly hypermethylated in the CHH context.

### Nodule Developmental Stages are Associated With Common and Unique Sets of Differentially Methylated miRNAs

We compared the differentially methylated miRNAs identified at each time point in order to identify common and unique set of differentially methylated miRNAs. We identified 12, 25, and 23 miRNAs as uniquely differentially methylated at 12, 22, and 36 dpi. Only three miRNAs (gma-MIR2107, gma-MIR10191, and gma-MIR2108a) were common to the three time points (Figure 3F). Also, we found only two and three differentially methylated miRNAs common to the 12 dpi nodules and those of the 22 and 36 dpi, respectively, (Figure 3F). In contrast, 20 differentially methylated miRNAs were shared between the 22 and 36 dpi. These data suggest that DNA methylation may contribute to the regulation of miRNAs in common and nodule stage-specific manner.

### DMRs in miRNA Promoters Overlap With TEs of the Copia and Gypsy Families

Previous study has indicated that the occurrence of TEs in the promoter of the miRNAs may contribute to their vulnerability to DNA methylation changes (Rambani et al., 2020a). Therefore, we examined promoter-DMRs for the presence of TEs. Our analysis revealed that about one-third (25 miRNAs) of the 82 differentially methylated miRNAs contains 31 unique DMRs that overlap with TEs (Supplementary Table S4). These TEs belong mostly to the Copia and Gypsy families of class I retrotransposons. Interestingly, 18 of these 25 differentially methylated miRNAs were hypermethylated (Supplementary Table S4). These results indicate that TEs in the promoter of miRNA genes may contribute but not the main mechanism responsible for miRNA gain or loss of DNA methylation during nodule development.

### Impact of Promoter-DNA Methylation on the Expression of miRNA Genes

**FIGURE 4:**
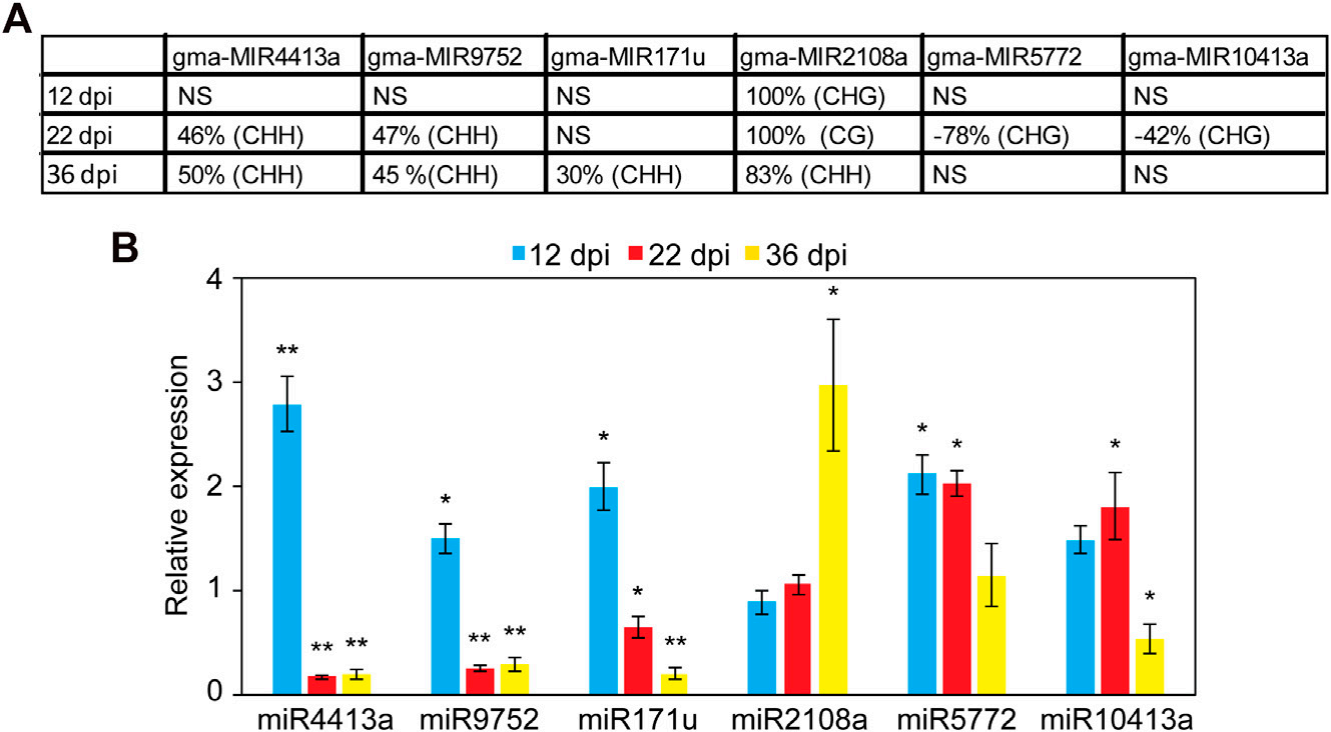
Analysis of the expression levels of promoter hyper- and hypomethylated miRNAs in nodules and root tissues. **(A)**: DNA methylation levels of six selected miRNAs showing hyper or hypomethylation only in the promoter region at different nodule developmental stages. Positive values indicate hypermethylation and negative values indicate hypomethylation. NS, not significant. **(B)**: Impact of promoter hyper and hypomethylation on the expression of miRNA genes in the developing nodules. The expression levels of miR2108a (hypermethylated at all time points), gma-miR4413a, gma-miR9752 (hypermethylated at 22 and 36 dpi), gam-miR171u (hypermethylated at 36 dpi), gma-miR5772, and gma-miR10413a (hypomethylated at 22 dpi) were quantified using qRT-PCR in nodules and roots tissues at 12, 22, and 36 dpi. Fold change values denote expression levels in nodules samples relative to the corresponding control root samples, which were set to 1. Data are average of three biologically independent samples ± SE. Statistically significant differences from the root control samples were calculated using t tests with *p* value <0.05 and indicated by asterisk.

We examined the impact of DNA methylation on the expression of miRNAs by quantifying the expression of six miRNA genes using quantitative real-time RT-PCR (qPCR). These miRNAs were selected because they were hyper or hypomethylated only in the promoter region at different time points. This includes gma-miR2108a (hypermethylated at all time points), gma-miR4413a, gma-miR9752 (hypermethylated at 22 and 36 dpi), gam-miR171u (hypermethylated at 36 dpi), gma-miR5772, and gma-miR10413a (hypomethylated at 22 dpi) (Figure 4A). Also, no TE was found to overlap with the DMRs identified in the promoter of these miRNAs with the exception of gma-miR9752, which contains a TE from the Copia family that overlaps with the identified DMR (Chr15:44640718–44650154; Supplementary Table S4). Nodules and root tissues were collected from several plants in three biological samples at 12, 22, and 36 dpi and used for RNA extraction and qPCR analysis. Consistent with the repressive effects of DNA methylation on gene expression, miR4413a and miR9752, which were hypermethylated in the 22 and 36 days-old nodules, showed significant downregulation in the nodules at both time points as compared with the corresponding control root tissues (Figure 4B). Similarly, miR171u, which was hypermethylated in the 36 days-old nodules, also exhibited significant downregulation in the nodules at the same time points as compared with roots (Figure 4B). gma-miR2108a, which was hypermethylated at all time points, however, showed significant upregulation at the 36-dpi time point (Figure 4B). We also examined whether loss of DNA methylation would result in increased miRNA expression. Interestingly, miR5772 and miR10413a, which showed hypomethylation in the promoter region in the 22-day-old nodules showed significant increases of about two-fold in the nodules relative to the corresponding control root tissues (Figure 4B). These data imply that gain and loss of DNA methylation in miRNA gene promoter contribute to miRNA gene expression changes in the nodules.

### Target Identification of Differentially Methylated miRNAs

We next analyzed previously published degradome-sequencing datasets (Fang et al., 2013; Hu et al., 2013; Arikit et al., 2014; Yan et al., 2015; Zhao et al., 2015; Ding et al., 2016; Xu et al., 2016) to identify target genes for the 82 differentially methylated miRNAs. Target genes for these miRNAs were also predicted by identifying perfect or near-perfect complementarity sequences of the mature miRNA in gene transcripts using psRNATarget web server (Dai et al., 2018). We detected 206, 496 and 537 target genes for the differentially methylated miRNAs identified at 12, 22, and 36 dpi, respectively, (Supplementary Tables S5–S7). We compared the target gene lists with our lists of differentially expressed genes identified in the nodules at the same three time points (Niyikiza et al., 2020). We found that 28% (58 genes), 38% (186 genes) and 39% (209 genes) of the identified targets at 12, 22, and 36 dpi were significantly differentially expressed at the corresponding time points. These data suggest that DNA methylation status of miRNAs can impact their expression and indirectly the expression of a large number of target genes in the nodules.

**FIGURE 5:**
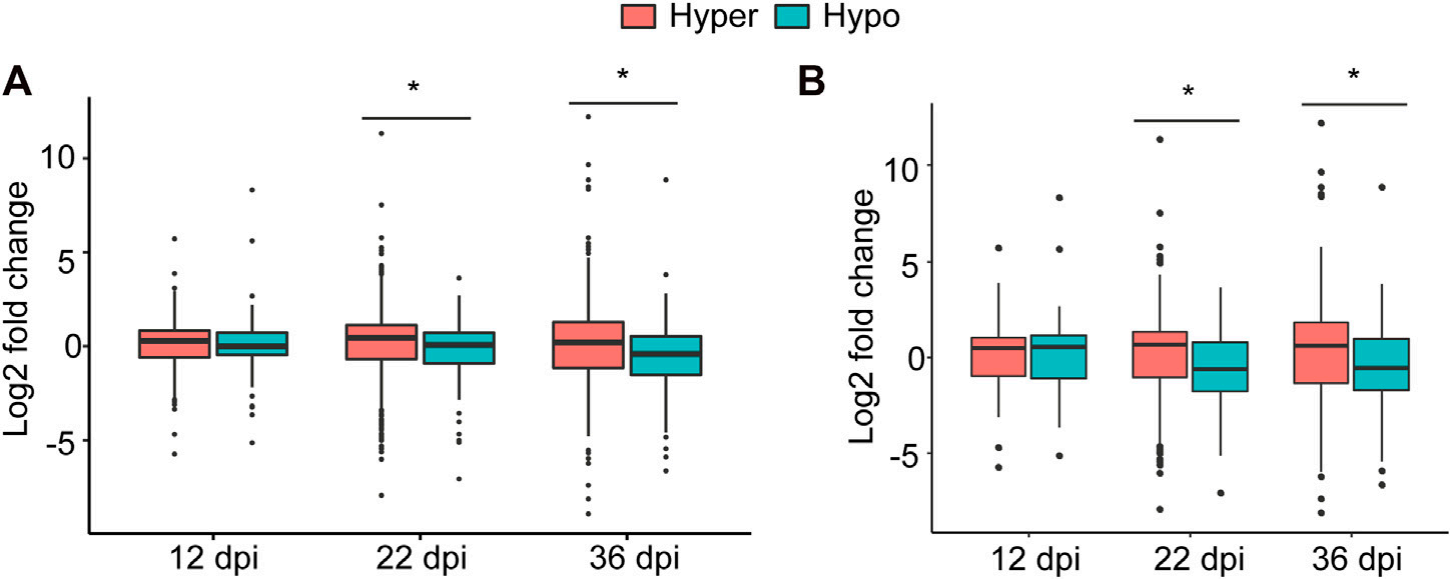
Impact of DNA methylation of miRNAs on target gene expression. **(A)**: Boxplot comparing the expression levels (regardless of the statistical significance) of all target genes of hyper- and hypomethylated miRNAs identified at 12, 22, and 36 dpi. **(B)**: Boxplot comparing the expression levels of only statistically differentially expressed target genes of hyper and hypomethylated miRNAs identified at 12, 22, and 36 dpi. Asterisks indicate statistically significant difference (*p* < 0.05) as determined by *t* test.

To examine this possibility, we associated hyper- and hypomethylated miRNAs with the expression of the identified target genes at each time point that were retrieved from our RNA-seq data (Niyikiza et al., 2020). While no significant association between methylation direction of the differentially methylated miRNAs and the expression of their targets at 12 dpi was found, statistically significant associations at 22 and 36 dpi were detected (Figure 5A). More specifically, the expression levels of genes targeted by hypermethylated miRNAs were statistically significantly higher than those genes targeted by hypomethylated miRNAs at both time point (Figure 5A). When we performed this analysis using only target genes that were differentially expressed between nodule and root tissues, we obtained similar results (Figure 5B). Together, these data imply that DNA methylation of miRNAs affect the expression of their target genes particularly during nodule development (22 dpi) and early senescence stages (36 dpi).

### Target Genes of Differentially Methylated miRNAs Are Involved in Nodulation

**FIGURE 6:**
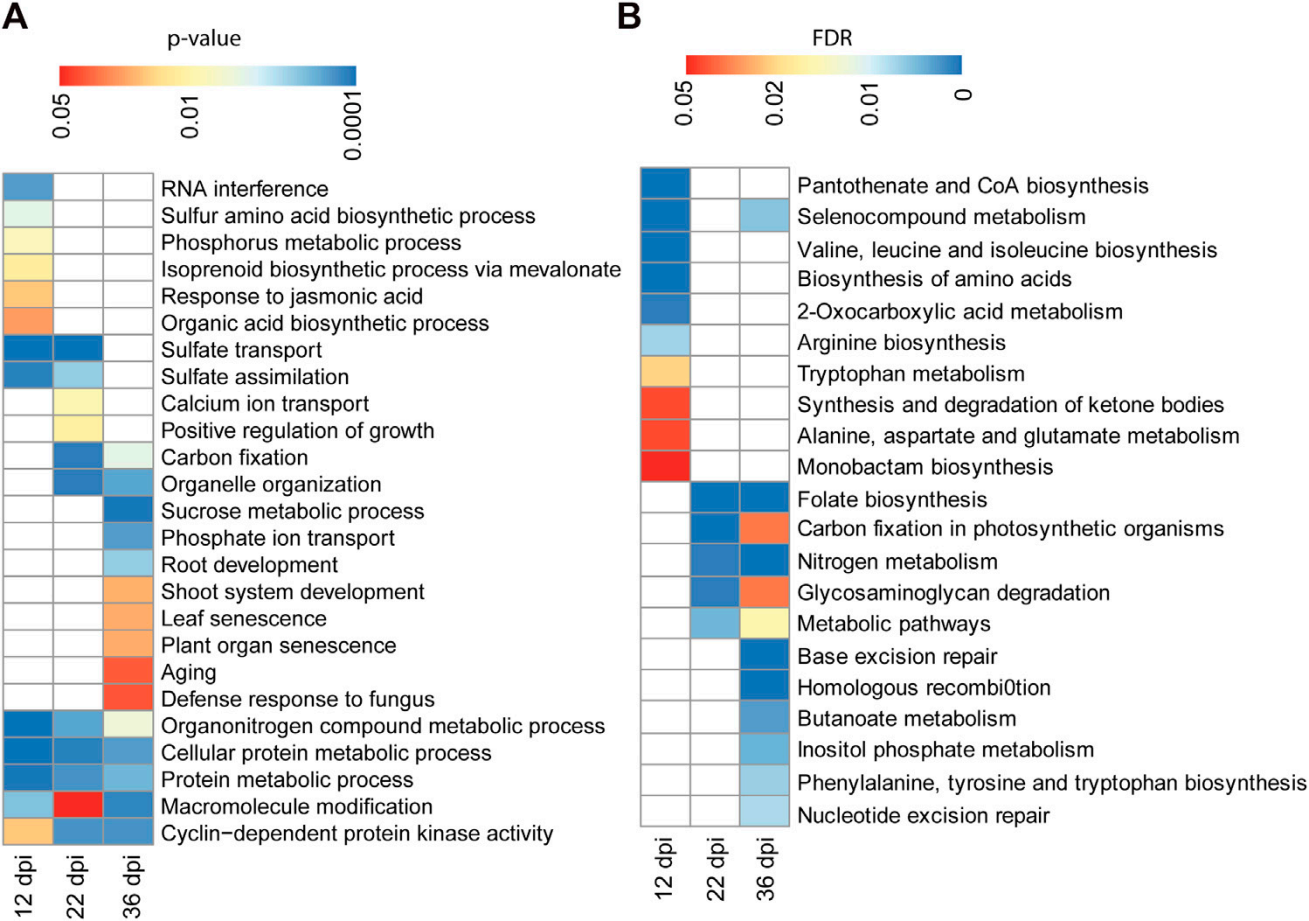
GO term and pathway analysis for the identified targets of differentially methylated miRNAs. **(A)**: GO term enrichment analysis for 206, 496, and 537 target genes of the differentially methylated miRNAs identified at 12, 22, and 36 dpi, respectively. Enrichment analysis was performed using Fisher’s exact test with *p* < 0.05. **(B)**: KEGG pathway analysis for 206, 496, and 537 target genes of the differentially methylated miRNAs identified at 12, 22, and 36 dpi, respectively. Enrichment analysis was performed using Fisher’s exact test and Benjamini and Hochberg FDR <0.05.

We performed Gene Ontology (GO) classification and enrichment analyses of the identified targets of differentially methylated miRNA genes at each time points. Targets of the 12 dpi differentially methylated miRNAs were enriched in biological process GO terms associated with sulfur amino acid biosynthetic process, phosphorus metabolic process, response to jasmonic acid and isoprenoid biosynthetic process (Figure 6A). Likewise, targets of miRNAs that are differentially methylated at 22 dpi were enriched in biological processes associated with calcium ion transport and regulation of growth (Figure 6A). GO terms associated with biological processes such as sucrose metabolic process, phosphate ion transport, plant organ senescence and aging were enriched among the putative targets of the 36 dpi differentially methylated miRNAs (Figure 6A). Genes involved in sulfate transport and assimilation are enriched among the targets of differentially methylated miRNAs at 12 or 22 dpi. GO term associated with organonitrogen compound metabolic process was enriched among the targets of miRNAs that are differentially methylated at 12, 22, or 36 dpi (Figure 6A).

We also performed KEGG (Kyoto Encyclopedia of Genes and Genomes (KEGG) pathway analysis to assign the targets of differentially methylated miRNAs to metabolic pathways. Targets of the 12 dpi differentially methylated miRNAs were enriched for pathways related to amino acid biosynthesis (Figure 6B). Targets of the 22 and 36 dpi differentially methylated miRNAs were both enriched for pathways related to nitrogen metabolism, folate biosynthesis, and carbon fixation (Figure 6B), consistent with the importance of these pathways for nitrogen fixation during nodulation.

## Discussion

Recent studies have revealed the key regulatory role of DNA methylation during nodulation (Satgé et al., 2016; Niyikiza et al., 2020). In this study, we examined the importance of DNA methylation of miRNA genes during various stages of nodule development in soybean. Single-base resolution of bisulfite sequencing data revealed dramatic increase in global DNA methylation levels over the promoter and primary transcript regions of annotated miRNA genes in the soybean genome. The accumulative increases of DNA methylation levels were found in all sequence contexts and mirror DNA hypermethylation patterns detected in protein-coding genes in various nodule developmental stages (Niyikiza et al., 2020). However, the promoters of miRNA genes seem to be more vulnerable to DNA methylation changes compared with the promoters of protein-coding genes. While 3.73% of soybean protein-coding gene promoters were found to be differentially methylated in the nodules compared to root tissues (Niyikiza et al., 2020), our current analysis revealed that about 12% of the annotated miRNA gene promoters were differentially methylated in the nodules as compared to root tissues. Consistent with the observed global increases of DNA methylation, more than two-thirds of the differentially methylated miRNAs were hypermethylated particularly in the CHH context. Substantial increases in the CHH-context methylation were observed in the nodules of soybean and Medicago (Satgé et al., 2016; Niyikiza et al., 2020), root *columella* cells (Kawakatsu et al., 2016) and during seed development (Kawakatsu et al., 2017) and fruit ripening (Lang et al., 2017; Huang et al., 2018). Therefore, it is tempting to speculate a role of CHH methylation in cellular differentiation and organ development despite direct evidence remains lacking.

Increased DNA methylation levels of miRNAs during nodule development are probably the result of increased expression of *DRM2* and its homologs accompanied by decreased expression of the demethylases *ROS1* and *DME* in the developing nodules compared to root tissues as recently reported by Niyikiza et al. (2020). Our finding that 26 miRNAs were hypomethylated in the nodules compared with roots indicates that methylation changes are dynamic and occur in both directions at different magnitudes through the activity of both DNA methyltransferase and demethylase enzymes. A recent study has revealed that the presence of TEs in miRNA gene promoter may contribute to miRNA susceptibility to DNA methylation changes (Rambani et al., 2020a). In agreement with this study, we found that about one-third of the DMRs located in the promoter region overlapped with TEs mainly of the Gypsy and Copia families. This is consistent with the findings that Gypsy and Copia are the most prevalent differentially methylated TEs in soybean nodules (Niyikiza et al., 2020), and tend to be more accessible to DNA methylation changes compared with other TE families (Hewezi et al., 2017; Rambani et al., 2020b). Consistent with a previous report that miRNAs encoding genes are primarily intergenic (Turner et al., 2012), we found that 77 of the 82 differentially methylated miRNA genes in the intergenic region and only five miRNA genes (gma-MIR1520m, gma-MIR5777, gma-MIR1520d, gma-MIR171u, and gma-MIR4354) in the body of protein-coding genes. This finding points into a possible co-regulation between these five miRNAs and their parental protein-coding genes.

The findings that substantial changes in DNA methylation occur during pathogen infection, cellular differentiation and organ development (Ikeuchi et al., 2015; Kawakatsu et al., 2016, 2017; Hewezi et al., 2018; Huang et al., 2019; Niyikiza et al., 2020), suggest that dynamic reprogramming of DNA methylation levels of miRNAs is important for nodule development. Several lines of evidence revealed the biological significance of DNA methylation in miRNA promoter and primary transcript regions during soybean nodulation. First, qPCR quantification of the expression levels of a number of miRNAs revealed an association between DNA methylation and miRNA expression. Though DNA methylation is mainly considered as a transcriptionally repressive epigenetic mark, our qPCR results pointed into a role of DNA methylation in gene activation. An association of DNA methylation and gene activation has been previously reported (He et al., 2011; Rambani et al., 2015; Lang et al., 2017; Huang et al., 2019; Gallego‐Bartolomé, 2020; Rambani et al., 2020b). Gene activation can be mediated through transcription factors that bind directly to methylated DNA motifs or through their associations with methyl-CpG-binding domain proteins (Zhu et al., 2016). Also, DNA methylation in gene promoter may enhance gene expression by preventing cryptic transcription initiation from TEs located in gene promoter that may interfere with proper gene transcription (Le et al., 2020).

Second, the target genes of hypermethylated miRNAs were expressed at significantly higher level than the targets of hypomethylated miRNAs at 22 and 36 dpi. This is consistent with the global inhibitory effects of DNA methylation on miRNA expression that result in increases in transcript abundance of the target genes. Our finding that 453 targets of the differentially methylated miRNAs significantly change the expression in the developing nodules as compared with roots suggests that miRNA methylation causally impacts gene expression during nodulation.

Third, miRNA target genes included transcription factors belonging to the MYB, bHLH and GRAS families, as well as those encoding nitrate transporters, nodulins, and components related to biosynthesis and signaling of phytohormones such as auxin, cytokinin, jasmonic acid, and salicylic acid whose role in nodule formation and functions are well established (Udvardi et al., 2007; Libault et al., 2009a, 2009b; Chiasson et al., 2014; Clarke et al., 2014; Ferguson and Mathesius, 2014; Bensmihen, 2015). Importantly, the target genes were enriched in biological processes related to nodule development and functions such as nitrogen metabolism, sulfur metabolism and transport, phosphorous metabolism, and calcium transport (Amor et al., 2003; Sulieman and Tran, 2015; Becana et al., 2018; Liu et al., 2018). We also observed that target genes of differentially methylated miRNA at the early stage of nodule senescence (36 dpi) were enriched in GO terms associated with organ senescence, aging, and defense response. Together, GO term and pathways analyses indicate that changes in DNA methylation of miRNA genes are not random but rather reprogrammed and contribute to nodule formation and development through indirect regulation of genes associated with cellular processes and pathways involved in nodulation.

Finally, it may be important to mention that several members of the identified differentially methylated miRNA families such as gma-MIR164, gma-MIR166, gma-MIR171, gma-MIR393, gma-miR2606, and gma-miR4416 have been functionally characterized and found to play vital role in nodule development (Subramanian et al., 2008; Branscheid et al., 2011; D’haeseleer et al., 2011; Yan et al., 2015). For example, constitutive overexpression of soybean gma-miR2606b and gma-miR4416 resulted in increasing nodule number (Yan et al., 2016). Together, these results suggest that the identified differentially methylated miRNAs and their targets play important roles in soybean nodule formation and development.

## Data Availability

The datasets presented in this study can be found in online repositories. The names of the repository/repositories and accession number can be found in the article/Supplementary Material.
